# The Effects of Comorbidity on the Benefits and Harms of Treatment for Chronic Disease: A Systematic Review

**DOI:** 10.1371/journal.pone.0112593

**Published:** 2014-11-17

**Authors:** Terri R. Fried, John O’Leary, Virginia Towle, Mary K. Goldstein, Mark Trentelange, Deanna K. Martin

**Affiliations:** 1 Clinical Epidemiology Research Center, VA Connecticut Healthcare System, West Haven, Connecticut, United States of America; 2 Department of Medicine, Yale School of Medicine, New Haven, Connecticut, United States of America; 3 Program on Aging, Yale School of Medicine, New Haven, Connecticut, United States of America; 4 Palo Alto Geriatrics Research Education and Clinical Center (GRECC), VA Palo Alto Health Care System, Palo Alto, Connecticut, 94304, United States of America; 5 Center for Primary Care and Outcomes Research, Stanford University School of Medicine, Stanford, California, 94305, United States of America; Leiden University Medical Centre, Netherlands

## Abstract

**Background:**

There are concerns about the potential for unintentional harms when clinical practice guidelines are applied to patients with multimorbidity. The objective was to summarize the evidence regarding the effect(s) of comorbidity on the outcomes of medication for an index chronic condition.

**Methods:**

A systematic review was conducted of studies published in MEDLINE and Cochrane Trials before May 2012. The search strategy was constructed to identify articles indexed with “comorbidity” or a related term or by a given condition and one or more additional specified comorbid conditions. The search yielded 3252 articles, of which 37 passed the title/abstract screening process, and 22 were included after full-text review. An additional 23 articles were identified by screening the reference lists for included articles. Information was extracted on study design; population; therapy; comparison groups; outcome(s); main findings.

**Findings:**

Indexing of articles was inconsistent, with no term for “multimorbidity,” and rare use of “comorbidity”. Only one article examined the effects of comorbidity *per se,* finding no benefit of tight control of DM among persons with high comorbidity, defined using a comorbidity index. The remainder examined pairs of conditions, the majority of which were post-hoc analyses of randomized controlled trials and which found no difference in outcomes according to whether a comorbid condition was present. Several demonstrated no difference or an increased risk of adverse outcome among persons with DM and tight control of HTN as compared to usual control. Several demonstrated lack of benefit of statins among persons with end-stage renal disease.

**Conclusions:**

There is limited evidence regarding the effects of multiple comorbidities on treatment outcomes. The majority of studies demonstrated no effect of a single comorbid condition on outcomes. Additional studies examining a broad range of comorbidity are required, along with clear and consistent indexing to allow for improved synthesis of the evidence.

## Introduction

Multimorbidity, or the co-existence of multiple diseases, is the most common chronic condition among adults. [1] There is increasing concern about the appropriateness of disease-based clinical practice guidelines to the care of the patient with multimorbidity. When applied to a theoretical patient, the many medications recommended by these guidelines result in the potential for adverse drug-drug and drug-disease interactions. [2] The likelihood of adverse drug events increases with the number of medications, [3] leading to questions about altered benefit/harm ratios associated with the prescription of multiple medications for patients with multimorbidity. [4] In addition, evidence about the benefits and harms of interventions from randomized controlled trials (RCTs) may not be generalizable to patients with multimorbidity. These patients are frequently excluded from the trials that form the evidence base for practice guidelines, and the trials have incomplete ascertainment of harms [4].

We sought to determine the extent of the evidence regarding the treatment of persons with multimorbidity by performing a systematic literature review. Although RCTs are generally considered to provide the highest quality evidence regarding treatment benefits, we elected to include both RCTs and observational studies because of the limitations of RCTs as applied to this population of patients and because of evidence that rigorously conducted observational studies provide accurate estimates of treatment effects [5].

## Materials and Methods

### Data Sources and Searches

Because “multimorbidity” is not a MeSH terms, the search was constructed around the concept of “comorbidity” to address the following question: “Among persons age 65 years and older, what effect does the presence of comorbid conditions have on the benefits and/or harms of medical treatment for a specific index chronic condition?” The following databases were searched for relevant studies: MEDLINE (OvidSP 1946–May Week 5 2012, April 13, 2012); MEDLINE In-Process & Other Non-Indexed Citations (OvidSP, April 13, 2012); Cochrane Trials (Wiley, April 11, 2012). While there are medical subject headings (MeSH) for comorbidity, when we examined four key articles, we found that only one of the four articles was indexed using these terms. We therefore had to create search strategies to capture the concept of comorbidity. We did this by using search terms that consisted of a given condition and at least one other of a list of additional conditions (heart failure [HF], coronary artery disease [CAD], angina pectoris, stable angina, hypertension [HTN], chronic obstructive pulmonary disease, osteoarthritis, hyperlipidemias, chronic kidney disease [CKD], diabetes mellitus [DM]). We repeated this process, with each condition becoming the index condition, and the rest considered together as comorbid conditions, using controlled vocabulary terms and synonymous free text words. The search strategy was limited to cohort studies, randomized controlled trials, or decision support techniques (Appendix S1). We specified older age in our question because of the increasing prevalence of multimorbidity with age, [6] with the goal of identifying study populations with the broadest possible range of comorbid conditions.

Because of the large number of references resulting from this search, the first 100 abstracts were reviewed to determine whether the search strategy could be made more specific. We found that we had captured a large number of studies that examined appropriateness of a given therapy, with appropriateness determined according to whether the patient had a given condition rather than according to the outcome of therapy. We therefore reran the analysis excluding these studies. Because of our interest in medical therapies, we also excluded studies that focused on surgical interventions. The full strategy is shown in appendix S1.

### Study Selection

We included articles that examined the outcomes of treatment for an index condition in the presence and absence of comorbidity. We also included articles that examined the outcomes associated with different intensity of treatment of an index condition in the presence of comorbidity, regardless of whether the treatment was explicitly defined. For example, we included articles examining outcomes according to different HgbA1C levels, regardless of whether the diabetic treatment was specified. We included articles that examined only the effect of a treatment for a given condition in the presence of comorbidity (e.g. there were no participants without the comorbid condition) when the effect of the treatment in the absence of the comorbidity is well established.

We excluded articles that examined surrogate outcomes, such as lab values. This exclusion was based on evidence that surrogate outcomes are frequently not accurate markers for clinically relevant outcomes and that studies examining surrogate outcomes may not capture relevant harms. [7] We did not exclude any clinically relevant outcomes, and planned to include studies examining quality of life outcomes, such as symptoms and function. In addition, it became apparent that the search strategy resulted in many articles with persons under the age of 65. Articles were therefore included if the study population included persons age 65 years or older, even if they also included younger persons. A total of 50 titles and/or abstracts were reviewed independently by three of the investigators (TRF, JRO, VT) to confirm uniformity in the process of excluding articles. The titles and/or abstracts of the remaining references were initially reviewed by one of two investigators (JRO, VT), and articles that were not excluded were re-reviewed by a third investigator (TRF) to achieve consensus regarding inclusion. Full text review was performed for these articles. The reference lists for the final set of articles identified by this search were also examined to identify additional articles meeting inclusion criterion.

### Data Extraction and Quality Assessment

For each of the included studies, we extracted the following data elements: 1) study design; 2) study population, including index condition; 3) intervention; 4) comparison groups; 5) outcome; 6) main findings.

We did not perform quality assessments for the articles based on two considerations. First, for the reasons provided above, we included both RCTs and observational studies. This posed a challenge for doing quality assessment, since, on the one hand, observational studies are generally considered to provide lower quality evidence than are randomized controlled trials, [8] but, as discussed above, in the case of multimorbidity, randomized controlled trials may not enroll patients with representative comorbidity burden. It is therefore difficult to apply metrics across the two types of study design that reflect their relative quality as specifically related to the study question. Second, many studies consisted of post-hoc analyses of RCTs, so that the initial randomization no longer applied, and there are no established criteria to evaluate the extent to which this affects the quality of the study. Given these considerations, we concluded that the best representation of study quality was a description of the study design, as extracted from each of the studies.

### Data Synthesis and Analysis

Because the large majority of studies examined treatment for an index condition among persons with or without a single comorbid condition, we organized studies according to the pairs of conditions being examined. Even within these groups, the heterogeneity in design, population, and interventions precluded combining the results in a meta-analysis.

## Results

### Identification of Articles

**Figure 1:**
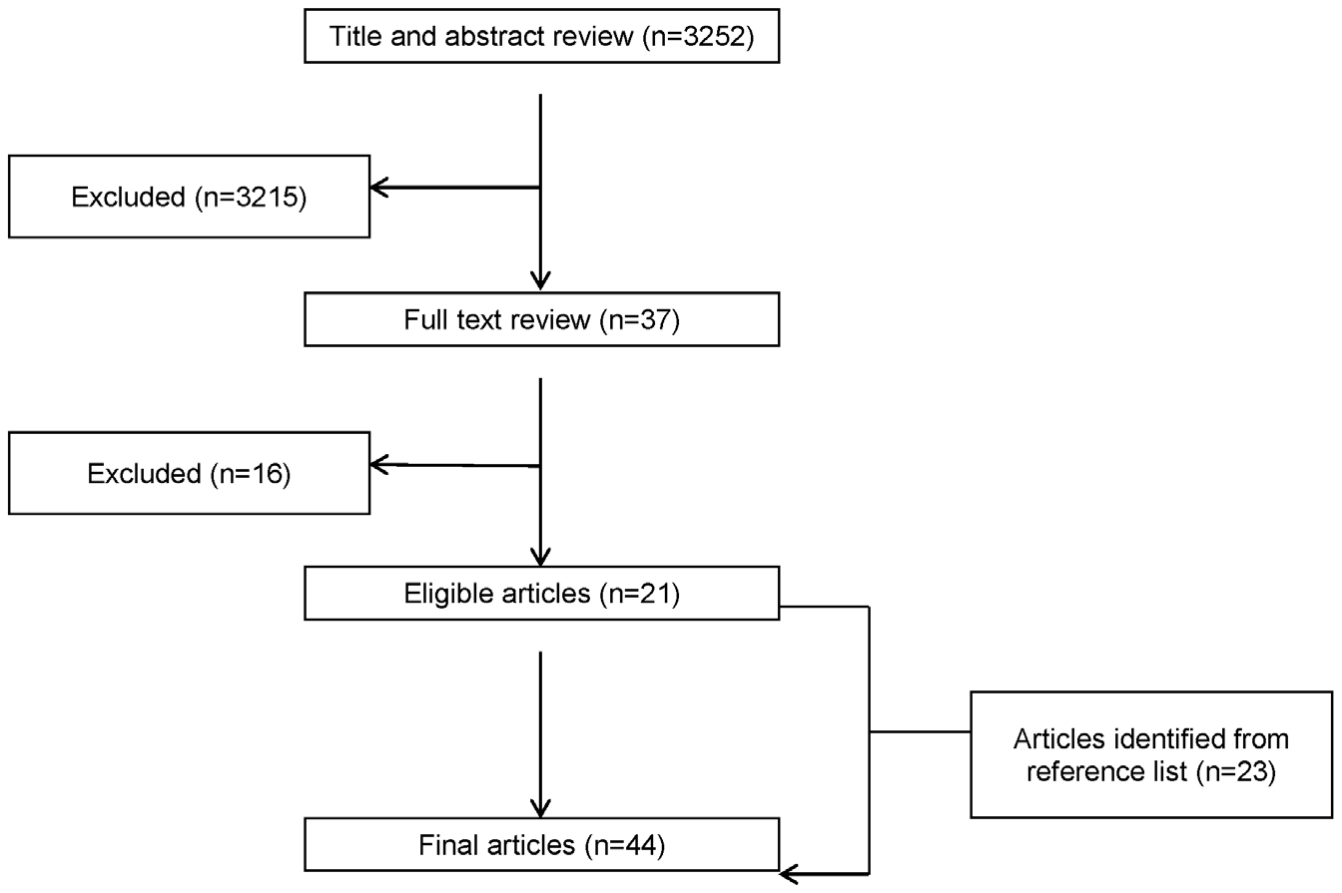
Summary of literature search and selection.

The literature search yielded 3252 articles. A total of 38 articles passed the title/abstract screening process. Full-text review resulted in the exclusion of 16 articles. Review of the reference lists for the remaining 22 articles resulted in the identification of an addition 23 articles, for a total of 45 (Figure 1).

### Types of Studies Identified

Table 1 provides a description of the types of studies identified in the systematic review, including the approach to the analysis of comorbidity, conditions and outcomes examined, and study design. Only one of the studies examined the effect of comorbidity *per se*. [9] This study examined the cardiovascular outcomes associated with tight diabetic control among persons with different levels of comorbidity. The remaining 44 studies examined the effects of treating an index condition in the presence or absence of a single other condition. The vast majority of these studies included DM as either the index or comorbid condition. The most common combinations of conditions examined were HF and DM (10 studies), [10]–[19] HTN and DM (9 studies), [20]–[28] and DM and CKD (10 studies) [29]–[38]. The studies examining HF and DM focused primarily on all-cause mortality among patients with HF who received treatment with beta blockers according to the presence or absence of DM. The majority of studies examining HTN and DM compared outcomes according to different intensities of HTN treatment, measured as the desired or achieved systolic and/or diastolic blood pressure. The studies examining CKD and DM primarily compared outcomes according to different intensities of diabetes treatment, measured as the achieved HgbA1C. An additional 5 studies examined DM and cardiovascular disease other than HF; these studies varied in terms of which was considered the index condition and which the comorbidity, the interventions, and outcomes [39]–[43].

**Table 1:** Description of studies identified in systematic review.

Approach to analysis of comorbidity:	
Comorbidity index	1
Pairwise combinations of conditions	44
Combinations of conditions*:	
Heart failure and diabetes mellitus	10
Diabetes mellitus and hypertension	9
Cardiovascular disease and diabetes	5
Diabetes mellitus and renal disease	10
Cardiovascular disease and chronic kidney disease	8
Study design:	
Randomized controlled trial	6
Randomized controlled trial with post-hocsubgroup analysis	25
Observational cohort study	14
Miscellaneous combinations	5
Outcome(s) examined*:	
Mortality	35
Disease-specific outcome (e.g. stroke,cardiovascular event)	24
Hospitalization	5
Function/symptoms	1
Adverse effect	2

* Total >45 because some articles examined more than one combination of conditions and/or more than one outcome.

A total of eight studies examined cardiovascular disease (HF, ischemic heart disease, or hyperlipidemia) and CKD [14], [44]–[50]. A total of four studies examined a variety of miscellaneous disease combinations [41], [51]–[53].

Across the different disease combinations, over one-half (55%) were conducted as post-hoc analyses of randomized controlled trials, in which patients with and without the comorbid condition were identified after study randomization, and the effect of the treatment was compared in these two groups. There were two studies that included only patients with both the index condition of hyperlipidemia or high cardiovascular disease risk and the comorbidity of CKD. These were randomized controlled trials in which patients were assigned to treatment with a statin versus a placebo. These studies assumed a well-established benefit of statins among patients with the index condition who did not have CKD. Only two studies examined differences in the likelihood of harm according to whether a comorbid condition was present; the remainder of the studies examined the likelihood of benefit. Of the studies examining benefit, only one examined functional outcomes; the remainder examined reduction in risk of mortality, hospitalization, or disease-specific outcomes, such as cardiovascular events.

### Study Findings

The majority of studies found no effect of comorbidity on the benefits or harms of therapy for an index condition. Of the 25 studies examining the benefit of therapy for a given condition according to whether comorbidity was present or absent, 23 found no difference or a greater benefit in the presence of the comorbidity (Table 2). The two studies examining the harm of therapy according to whether comorbidity was present or absent found no difference in harms. Results were more mixed for the 15 studies examining the benefit of tighter versus less tight control of one condition in the presence of a second. Of these, seven found tighter control was more beneficial than less tight control, six found tighter control was of no greater benefit or was harmful compared to less tight control, one found a U-shaped relationship, and one had different findings for different outcomes.

**Table 2:** Main study findings according to approach of article.

Examination of benefit of therapy in presence or absence of comorbidity (n = 25):	
No difference in benefit	19
Benefit greater in presence of comorbidity	4
Benefit smaller or absent in presence of comorbidity	2
Examination of harm of therapy in presenceor absence of comorbidity (n = 2):	
No difference in harm	2
Harm smaller or absent in presence of comorbidity	0
Harm great in presence of comorbidity	0
Examination of benefit of tighter versus less tight control of onecondition when second condition present (n = 15):	
Tighter control more beneficial versus less tight control	7
Tighter control of no greater benefit or harmful versus less tight control	6
U-shaped relationship, or different relationship for different outcomes	2
Examination of benefit of intervention established as standard ofcare when comorbidity absent in the presence of comorbidity (n = 3):	
Intervention beneficial	1
Intervention not beneficial	2

Table 3 provides a detailed description of the study results according to the study population, study design, intervention(s) examined, and comparison groups. The single study examining comorbidity *per se* and its effects on the treatment of DM demonstrated that for persons with low to moderate comorbidity, measured with a validated comorbidity index, achieving a HgbA1C ≤6.5% was associated with a decreased risk of cardiovascular events, but for persons with high comorbidity, achieving this HgbA1C level was not associated with decreased risk [9].

**Table 3:** Studies identified in systematic review.

Author, Year	Study Design & N		Population	Intervention/Comparison Groups	Outcome/Main Findings(Differences between Groups)
**Diseases: HEART FAILURE AND DIABETES MELLITUS**					
Bobbio, 2003	Cohort N = 2,843		Italian patients w/HF,mean ages 63–66	Beta-blockers/with and w/o DM	All-cause mortality: no difference.
deBoer, 2010	Post-hoc RCTN = 2,128		Dutch patients ≥70 w/HF	Nebivolol (BB)/with and w/o DM	All-cause mortality and CV hospital admission: riskreduction greater in patients with DM.
Deedwania, 2005	Post-hoc RCTN = 3,991		European patients, ages40–80 w/HF	Metoprolol CR/XL (BB)/with and w/o DM	All-cause mortality and hospitalization due to HF: no difference
Domanski, 2003	Post-hoc RCTN = 2,708		US patients, ages 19–93 w/HF	Bucindolol (BB)/with and w/o DM.	Death or HF hospitalizations: no difference.
Erdmann, 2001	Post-hoc RCTN = 2,647		European patients w/HF,mean age 61	Bisoprostol/with and w/o DM	All-cause mortality: no difference.
MacDonald, 2008	Post-hoc RCTN = 7,599		European patients w/HF,mean ages 65–67	Candesartan/with and w/o DM	All-cause mortality, CV morbidity and mortality: no difference.
Nodari, 2003	CohortN = 193		Italian patients w/HF, mean ages 60 (w/DM)and 55 (w/o DM)	Carvedilol/with and w/o DM	NYHA functional class, exercise tests, and otherhemodynamic parameters: no difference.
Ryden, 2000	Post-hoc RCTN = 3,164		International patients >55 w/HF	Lisinopril high vs. low dose/with and w/o DM	All-cause mortality: no difference.
Subramanian 2009	CohortN = 412		Veterans w/HF, meanages 66–70	Beta-blockers (cardioselective CSB)/with and w/o DM	All-cause mortality: no benefit for patients with DM; borderline significant benefit for patients without DM.
Torp-Pedersen, 2007	Post-hoc RCTN = 3,029		European patients w/HF,mean ages 61–64	Carvedilol vs. metoprolol/with and w/o DM	All-cause mortality: no difference.
**Diseases: DIABETES AND HYPERTENSION**					
ACCORD, 2010 (Cushman)	Factorial RCTN = 4,733		DM pts ≥40 w/CVD or ≥55 w/atherosclerosis,albuminuria, LVH, or ≥2 risk factors for CVD	BP therapy/tight (<120 SBP) vs less tight (<140 SBP) BP control	Major cardiovascular event: no difference. All-cause orcardiovascular mortality: no difference Significantlyhigher rate SAE (3.3% vs. 1.29%, p<.001),hyperkalemia, and elevated Cr in tightcontrol group vs less tight group.
Berl, 2005	Post-hoc RCTN = 1,715		US patients, ages 30–70 w/DM, HTN, proteinuria	BP therapy/observed SBP≤120 vs >120; DBP 10 mm Hg increments	All-cause mortality: increased risk with SBP<120.MI: increased risk with lower DBP.Stroke: decreased risk with lower DBP.
Cooper-DeHoff, 2010	Post-hoc RCTN = 6,400		US patients, ≥50 w/stable CAD and DM	BP control/observed tight (SBP<130), usual (SBP 130–139), uncontrolled (≥140)	All-cause mortality: no difference during study f/u.5-yr mortality: higher in tight control.
Curb, 1996	Post-hoc RCTN = 4,736		US patients, ≥60 w/HTN	Chlorthalidone (+atenolol or reserpine as needed)/with and w/o DM	All-cause mortality, non-fatal plus fatal stroke, nonfatalMI plus fatal CHD, major CHD events, major CVD events:risk reduction as great if not greater for persons with DM(no formal test for interaction)
Estacio, 2000	RCT N = 470		US patients, ages 40–74 w/DM	Stepped BP therapy/DBP≤70 vs. DBP 80–89	CrCl, retinopathy, neuropathy: no difference.All-cause mortality: lower in tight control.
Hansson, 1998	Stratified RCTN = 1,501		European patients, ages 50–80 w/DM	Stepped BP therapy/DBP≤90 vs. ≤85 vs. ≤80	Major CV events: lower in tight control(significant difference ≤80 vs. ≤90).
Tuomilehto, 1999	Post-hoc RCTN = 4,695		European patients, ≥60 with HTN	Nitrendipine (+others as needed)/with and w/o DM	All-cause mortality, mortality fromcardiovascular disease, all cardiovascularevents: greater risk reduction in patients withDM
UKPDS, 1998	RCT N = 1,148		UK patients, ages 25–65 w/HTN & DM	BP therapy/tight (<150 SBP) vs. less tight (<180 SBP) BP control	All-cause mortality, MI: no difference. AnyDM-related endpoints, DM-related deaths,stroke, microvascular disease: lower in tight control.
Wang, 2000	Post-hoc RCTN = 2,394		Chinese patients, ≥60 w/HTN	Nitrendipine (+others as needed)/with and w/o DM	All-cause mortality, CV mortality, stroke,all cardiovascular events: no difference
**Diseases: CARDIOVASCULAR DISEASE AND DIABETES**					
Berthet, 2004	Post-hoc RCTN = 6,105		International patients w/hx of CVA or TIA,mean ages 63–64	ACE-inhibitor (+indapamide as needed)/with and w/o DM	Recurrent stroke: no difference.
Collins, 2003	Post-hoc RCTN = 20,536		UK patients, ages 40–80 w/CAD, PAD, or HTN.	Simvastatin/with and w/o DM	Coronary events and vascular events:no difference.
Komajda, 2010	Post-hoc RCTN = 4,447		International patients w/DM	Rosiglitazone/with and w/o CVD	Heart failure: no difference
Shinohara, 2008	Post-hoc RCTN = 1,095		Japanese patients w/prior CVA or TIA	Cilostazol/with and w/o DM, HTN	Recurrent stroke: no difference.
Wernicke, 2009	Post-hoc RCTs (pooled analysis)N = 1,024		US patients ≥18 with diabetic peripheral neuropathy	Duloxetine/with and w/o CVD	Adverse events: no difference.
**Diseases: DIABETES AND RENAL DISEASE**					
Hayashino, 2007	CohortN = 1,569	Japanese patients w/ESRD on HD, mean ages 58–64		Glycemic control during stable, regular HD/Quintile of HgbA1C	All-cause mortality: top quintile of HgbA1Cassoc w/increased risk compared to lowest quintile.
Kalantar-Zadeh, 2007	CohortN = 23,618	US patients w/ESRD on HD, mean ages 60–63		Glycemic control during stable regular HD/HgbA1C level	All-cause mortality: unadjusted analysis demonstrated lowerHR with higher HgbA1C; adjustment resulted inhigher HR as HgbA1c increased.
Lambers Heerspink, 2010	Post-hoc RCTN = 10,640	European patients with DM, mean age 65–68		Perindopril-indapamide/w/o CKD and w/3 stages of CKD	Macrovascular events, cardiovascular death, all-causemortality, cerebrovascular events, new or worseningnephropathy, renal death: no difference.
Mann, 2001	Post-hoc RCTN = 980	International patients >55 w/DM and cardiac risk factor		Ramipril/with and w/o CRI	CV death, MI or CVA (combined outcome): no difference.CV mortality, all-cause mortality, heart failure–relatedhospitalization: risk reduction greater in patients with CRI.
Morioka, 2001	CohortN = 150	Japanese patients w/ESRD on HD, ages 29–85, mean age 60.5		Glycemic control prior to start of dialysis/HgbA1C level	1, 3, and 5-year mortality: Risk increasedwith higher HgbA1C.
Okada, 2007	CohortN = 78	Japanese patients w/type 2 DM and ESRD, age ≥20,mean age 58		Glycemic control prior to start of dialysis and during dialysis/HgbA1C level	All-cause mortality: no difference.
Oomichi, 2006	CohortN = 114	Japanese patients w/ESRD on HD, ages 33–80, mean age 60.8		Glycemic control during stable, regular dialysis/Good, fair, poor HgbA1C	5-year mortalty: Higher in those w/poor HgbA1C (≥8)than in those with fair (6.5–8) or good (<6.5) HgbA1C.
Shurraw, 2010	CohortN = 1,484	Canadian patients receiving hemodialysis, mean age 66		Glycemic control/HgbA1C level	All-cause mortality: no difference.
Shurraw, 2011	CohortN = 23,296	Canadian patients w/DM and CKD, mean age 65–73		Glycemic control/HgbA1C level	All-cause mortality: U-shaped association:increased risk with <6.5% and >8.0%.
Williams, 2006	CohortN = 24,744	US patients w/ESRD on HD, mean age 63.7		Glycemic control during stable, regular dialysis/HgbA1C level	All-cause mortality: no difference.
**Diseases: CARDIOVASCULAR DISEASE AND CHRONIC KIDNEY DISEASE**					
Baigent, 2011	RCTN = 9,270	European patients, ≥40 with moderate-severe CKD		Simvastatin+ezetimibe vs. placebo*	17% decrease in major CVD events with treatment; nodifference in effect comparing patients with ESRD ondialysis and those with less severe CKD
Cohen-Solal, 2009	Post-hoc RCT (prespecified group)N = 2,112	European patients, >70 w/HF		Nebivolol/Tertiles of eGFR	All-cause mortality and CV hospitalizations:no difference.
Erdmann, 2001	Post-hoc RCTN = 2,647	European patients w/HF, mean age 61		Bisoprostol/with and w/o CKD.	All-cause mortality: no difference.
Fellström, 2009	RCTN = 2,776	European patients, ages 50–80 w/ESRD on HD		Rosuvastatin vs. placebo*	CV death, non-fatal MI, or non-fatal stroke: no risk reduction with rosuvastatin
McAlister, 2004	CohortN = 754	Canadian patients w/HF, median age 69		ACE-inhibitors and beta-blockers/with and w/o CKD (GFR <60)	All-cause mortality: no difference
Nakamura, 2009	Post-hoc RCTN = 7,195	Japanese patients, ages 40–70 w/HL		Pravastatin/with and w/o moderate CKD	CHD, stroke, CVD, all-cause mortality: risk reduction greater in moderate CKD than without moderate CKD.
Tonelli, 2004	Post-hoc RCTN = 19,700	International patients w/CAD or at high risk forCAD, mean ages 50–65		Pravastatin/with and w/o moderate CKD	MI, coronary death, PTCA/surgical revascularization: no difference.
Wanner, 2005	RCTN = 1,255	European patients, ages 18–80 w/Type II DM w/ESRD on HD		Atorvastatin vs. placebo*	Composite of cardiac death, nonfatal MI, and stroke: no risk reduction with atorvastatin
**MISCELLANEOUS DISEASE COMBINATIONS**					
Bavry, 2010	Post-hoc RCTN = 22,576	International patients, ≥50 w/HTN and stable CAD		BP lowering/with and w/o PAD	All-cause mortality, nonfatal MI and nonfatal CVA: more pronounced J-shaped curve for relationship of HTN and outcome among PAD compared to no PAD.
Du, 2009	Post-hoc RCTN = 11,140	International patients, ≥55 w/DM and 1 additional CV risk		Perindopril+indapamide/with and w/o AF	All-cause mortality, CV deaths, major coronary events, major cerebrovascular events, HF: no difference.
Greenfield, 2009	CohortN = 2,613	UK patients w/DM, mean ages 61–64		Glycemic control/w/low-to-mod comorbidity (TIBI <12) vs. high comorbidity (TIBI ≥12)	CV events: HbA1c ≤6.5 associated with reduced risk in low CM but not mod-high CM.
Shinohara, 2008	Post-hoc RCTN = 1,095	Japanese patients w/prior CVA or TIA		Cilostazol/with and w/o HTN	Recurrent stroke: no difference.
Sin, 2002	CohortN = 11,942	Canadian patients ≥65 w/HF		Beta-blockers/With and w/o IHD	All-cause mortality: no difference.

RCT = randomized controlled trial.

w/o = without.

HF = heart failure.

DM = diabetes mellitus.

CV = cardiovascular.

NYHA = New York Heart Association.

LVH = left ventricular hypertrophy.

CVD = cardiovascular disease.

BP = blood pressure.

HTN = hypertension.

SBP = systolic blood pressure.

SAE = serious adverse event.

DBP = diastolic blood pressure.

MI = myocardial infarction.

CAD = coronary artery disease.

CHD = coronary heart disease.

CrCl = creatinine clearance.

UK = United Kingdom.

ACE = angiotensin converting enzyme.

PAD = peripheral arterial disease.

CVA = cerebrovascular accident.

AF = atrial fibrillation.

IHD = ischemic heart disease.

ESRD = end = stage renal disease.

HD = hemodialysis.

CKD = chronic kidney disease.

PTCA = percutaneous transluminal coronary angioplasty.

TIBI = Total Illness Burden Index.

*No inclusion of patients without CKD and therefore no comparison between patients with and without CKD; study assumed established benefit of therapy in the absence of CKD.

**Figure 2:**
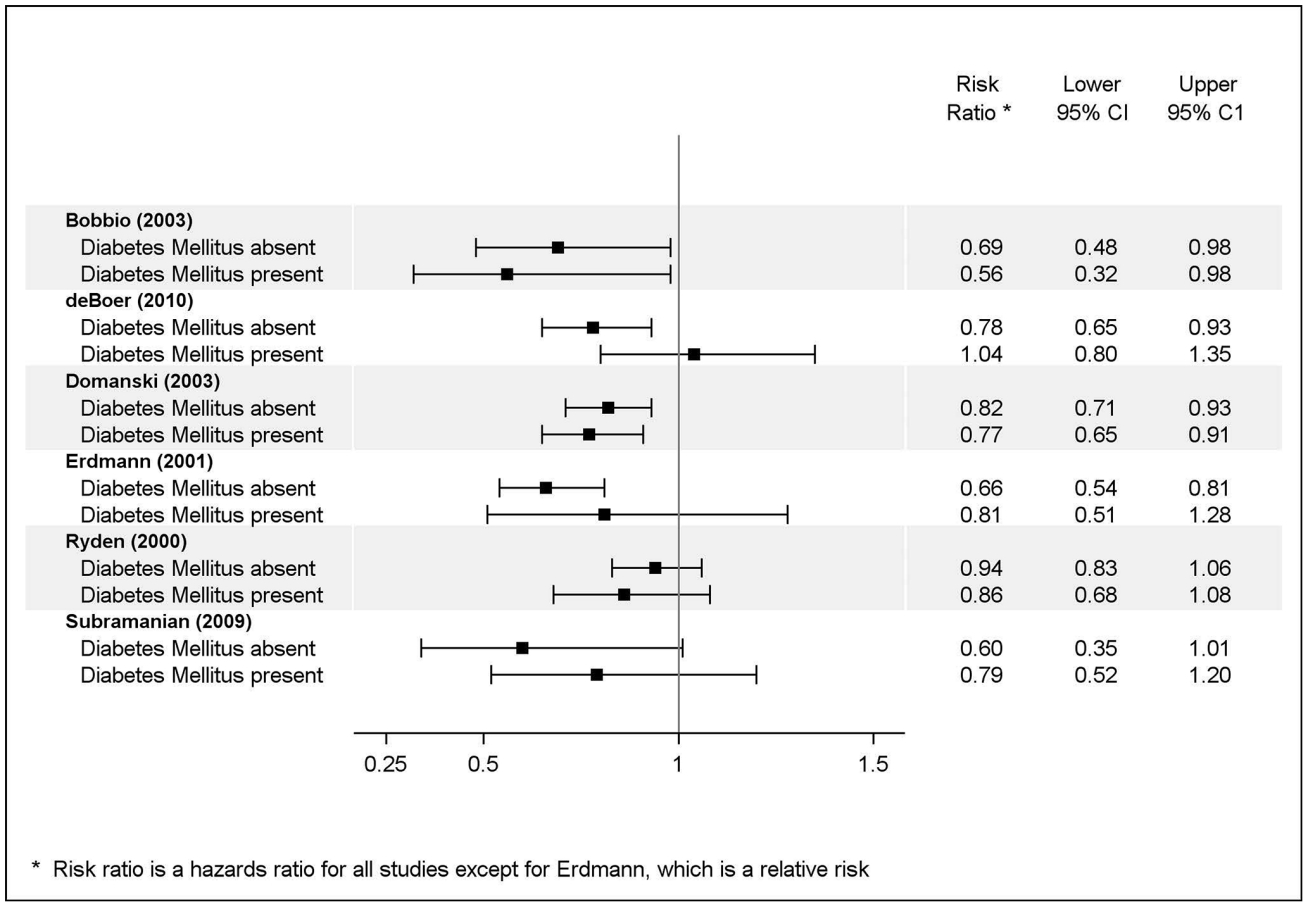
Risk Reduction Associated with Medication for Treatment of Heart Failure.

All but two of the ten studies examining the effect of DM on the treatment of HF with beta blockers concluded that there were no differences in outcomes according to the presence or absence of DM or greater risk reduction among patient with DM [10]–[17], [19]. Figure 2 provides a graphical summary of the results for those studies presenting their outcomes in terms of a form of relative risk. One study reported a significant benefit of beta-blockers on all-cause mortality and cardiovascular outcomes among patients without DM with a hazard ratio (HR) of.78 (95% confidence interval [CI].65, .93) but no benefit among patients with DM with a HR of 1.04 (.80, 1.35). [11] The second study concluding that there was a difference in all-cause mortality reported a non-significant effect of beta-blockers among patients with DM with a hazard ratio (HR) of.79 (95% confidence interval [CI].50, 1.20) and a borderline significant effect among patients without DM with a HR of.60 (.35, 1.01). [18] While another study also showed a significant effect among patients without DM and a non-significant effect among patients with DM, it concluded that there was no difference in benefit because of a non-significant “heterogeneity test for interaction.” [14].

Among the six studies examining intensity of HTN treatment in patients with DM, the definition of tight control changed over time, as did the study results. The United Kingdom Prospective Diabetes study (UKPDS), defining tight control as a systolic blood pressure (SBP) of <150 mmHg, versus less tight control as SBP<180 mmHg, demonstrated no difference in overall mortality associated with tight control but a decrease in any DM-related endpoints, DM-related deaths, stroke, or microvascular disease. [27] Later studies defining tight control as SBP<130 or <120 mmHg demonstrated either no difference in outcome or an increased risk of adverse outcome associated with tight control. [20], [21], [23] The results were less consistent when tight control was defined on the basis of diastolic blood pressure (DBP). Two studies defining tight control as DBP<80 mmHg and as <70 mmHg demonstrated benefits in terms of lower all-cause mortality and major cardiovascular events, [24], [25] whereas a third study examining DBP in 10 mm increments demonstrated a decreased risk of stroke but increased risk of myocardial infarction associated with tighter control [20].

Of the five studies examining patients with cardiovascular disease and diabetes, three examined the effects of medications to reduce risk of vascular outcomes (ACE-inhibitor, simvastatin, and cilostazol) and found no differences according to the presence or absence of DM [39]–[41]. The remaining two studies examined the likelihood of harm associated with medication use among persons with DM with and without cardiovascular disease. These studies found no difference in adverse effects associated with duloxetine and no difference in the likelihood of heart failure associated with rosiglitazone.

The eight studies examining outcomes in patients with renal disease according to control of blood sugar in DM showed conflicting results. A total of three studies demonstrated an increased risk of mortality associated with higher HgbA1C levels, [29], [33], [35] whereas three other studies demonstrated no difference in survival, [34], [37], [38] and one study demonstrated a U-shaped association. [36] In one study, the unadjusted analysis demonstrated a lower risk of mortality with higher HgbA1C levels but the adjusted analysis demonstrated a higher risk, suggesting that HgbA1C levels may be a marker of underlying health status. [30] The two studies examining the effects of ACE-inhibitors on cardiovascular outcomes among patients with DM demonstrated either no differences according to the presence or absence of CKD [31] or greater risk reduction among patients with renal disease [32].

**Figure 3:**
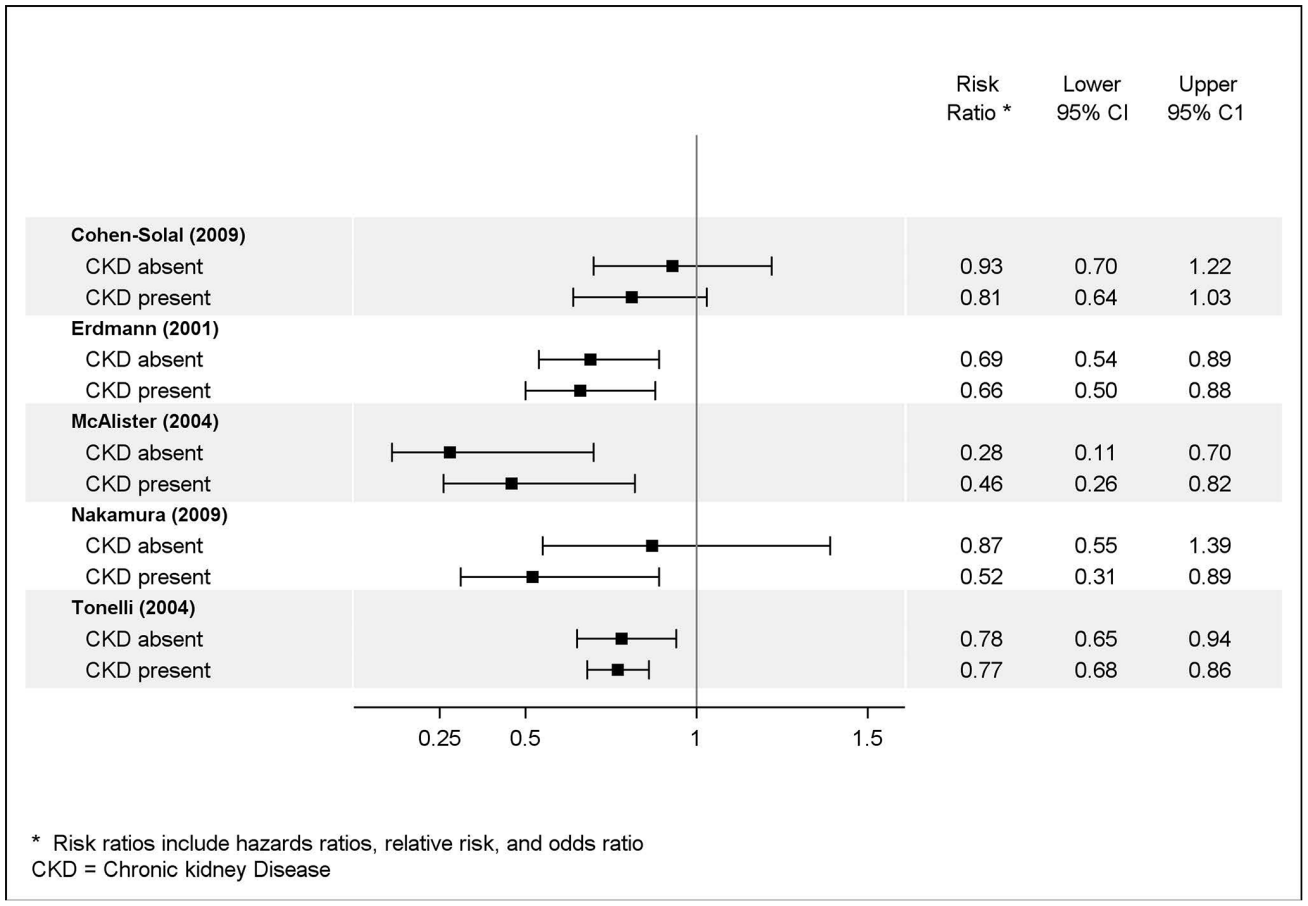
Risk Reduction Associated with Medication for Treatment of Cardiovascular Disease.

Of the five studies examining a variety of medications among patients with cardiovascular disease, four demonstrated no difference in outcomes according to the presence or absence of CKD. [14], [45], [47], [49] The fifth, a study of pravastatin, demonstrated a decreased risk of a number of different outcomes among persons with moderate CKD but no decreased risk among persons with normal renal function or mild CKD, suggesting increased efficacy of the medication in the presence of CKD. [48] (Figure 3). Of the three studies examining the efficacy of statins among persons with CKD without a comparison group of persons without CKD, one demonstrated a decreased risk of major cardiac events with treatment and found no difference when comparing patients with end-stage renal disease (ESRD) and those with less severe CKD. [44] The two other studies demonstrated no decreased risk of major cardio/cerebrovascular events with treatment among patients with ESRD receiving hemodialysis [46], [50].

Of the four studies examining miscellaneous combinations of conditions, three demonstrated no difference in the efficacy of treatment according to the presence or absence of the comorbid condition. This included the use of perindopril-indapamide among persons with DM and one additional cardiovascular risk factor in the presence or absence of AF, [52] the use of cilostazol in persons with prior cerebrovascular accident or transient ischemic attack in the presence or absence of HTN, [41] and the use of beta blockers in persons with HF in the presence or absence of ischemic heart disease. [53] The final study examined the effect of the intensity of treatment of HTN in persons with CAD in the presence or absence of PAD. This study found a J-shaped relationship between blood pressure and outcomes among persons with PAD, such that lower blood pressure was associated with increased risk, but no such relationship among persons without PAD [51].

## Discussion

This systematic review was undertaken to address the question of how the presence of comorbid conditions affects the benefits and harms of medical treatment for an index chronic condition. The process of the review revealed that there is virtually no evidence regarding the effect of comorbidity *per se*, as the review identified only one study that characterized patients according to their burden of comorbid illnesses. Instead, the evidence consists of studies of pairs of conditions, in which investigators examine the effect of therapy in the presence or absence of a single comorbid condition. There is also little evidence regarding harms of treatment, as the vast majority of studies focused on examining benefit only. In addition, benefit was narrowly defined in terms of reducing the risk of mortality and other major disease-specific outcomes. Studies focused on a limited range of pairs, consisting of a combination of cardiovascular disease (HF and HTN), DM, and CKD. Few studies were originally designed to address the question of the effect of comorbidity on treatment of an index condition. The majority of studies demonstrated that the presence of a comorbid condition did not reduce the benefits associated with treatment of the index condition. The exceptions to this were the findings that patients with a high burden of comorbidity do not derive the same benefit from tight diabetic control as patients with a lower comorbidity burden, that patients with DM do not benefit from tight control of HTN, and that patients with ESRD do not derive the same benefit from treatment with statin therapy as patients without ESRD.

This systematic review was motivated by concern raised in the literature about the unintended adverse consequences of treating patients with multimorbidity. This concern arises from the finding that treating patients with multimorbidity according to current disease-specific guidelines can result in polypharmacy with the potential for numerous drug-drug and drug-disease interactions. [2] Moreover, polypharmacy itself has been associated with adverse outcomes, including falls, [54] weight loss and balance impairment, [55] and hospitalization. [56] However, the existing literature does not address this patient population. Despite an extensive search, we found only one study that examined burden of comorbidity as a potential modifier of the effect of treatment. “Multimorbidity” is not an indexing term, and, while “comorbidity” is a term, it was infrequently used, so that it proved difficult to construct a search addressing our question of interest. With the growing attention being paid to the study of multiple medical conditions, there is a need for a clear and consistent approach to indexing future studies in order to facilitate the ability to synthesize evidence as it becomes available.

The great majority of studies examined either mortality or disease-specific outcomes, such as cardiovascular events. Disease-specific outcomes cannot be applied across individual diseases to facilitate treatment decision making that takes into account the net effect of an intervention on the health of a patient with multimorbidity, and universal outcomes that are applicable across diseases such as quality of life, physical function, or symptom reduction may be more important to these patients. [57], [58] Moreover, studies did not examine time to benefit, a parameter particularly important for patients with multimorbidity because of their competing mortality risks [57].

The studies conducted as post-hoc analyses of randomized controlled trials are subject to the same limitations of their patient population as the original studies. The patients eligible for and participating in cardiovascular intervention studies are generally healthier and with fewer comorbid conditions than are patients who do not enroll in these studies. [59], [60] There is evidence that these differences influence the benefits and harms of therapies. For example, the RALES RCT demonstrating the effectiveness of spironolactone in reducing hospitalization and mortality among patients with advanced HF had very low rates of adverse events. [61] The publication of the trial was followed by a marked increase in the use of spironolactone, and, among older persons who were taking ACE-inhibitors, this increase was accompanied by a substantial rise in the rates of hospitalization and mortality from hyperkalemia [62].

Because of the lack of studies examining comorbidity, our review ended up addressing the question of whether, among a limited spectrum of diseases, the presence of a second condition affects the benefits or harms of treatment for a first. While, for many pairs of conditions (DM and HF, DM and CKD, DM and CVD), the answer to this question appears to be “no,” there was some evidence to suggest that the presence of a second condition can alter the outcomes of therapy. The finding of an increased risk of adverse outcome among patients with DM receiving tight blood pressure control illustrates an evolution in the approach to the treatment of HTN among diabetic patients. Recommendations published in 2003 included the statement that “there is no threshold value for blood pressure, and risk continues to decrease well into the normal range,” [63] suggesting the lower the blood pressure, the better the outcomes. Recommendations published in 2013 call for treatment to a systolic blood pressure of <140, reserving treatment to lower targets for select populations, such as younger patients. [64] While these recommendations appear to be supported by the results of this systematic review, it is also worth noting that many disease management guidelines for DM also recommend different targets for HgbA1C according to comorbidities, ranging from 7% to 8%. Aside from the one study demonstrating a lack of benefit for patients with high comorbidity burden achieving a HgbA1C of <6.5%, our review did not identify any studies supporting these recommendations.

Our review has several limitations. First, because of the difficulty in constructing an effective search strategy, we found a relatively large proportion of our studies through review of the reference lists of articles identified in the search. This suggests that we may have missed relevant articles. Second data to inform our study question may exist that was not available in the articles we reviewed. For example, we identified two meta-analyses of studies examining the effect of DM on the treatment of HF. In our review of the articles referenced in the meta-analyses, we failed to find any subgroup analysis examining outcomes according to the presence or absence of DM. This suggests that the authors of the meta-analyses obtained additional data from the investigators of the original studies.

The conduct of this systematic review designed to address the question of how comorbid conditions affect the benefits and harms of treatment for an index chronic disease demonstrated that the evidence was difficult to find because of a lack of clear indexing. Moreover, few studies were designed to answer this question optimally. There was little evidence examining comorbidity *per se*, but rather studies examining pairs of conditions. Because many studies were derived from RCTs, their participants may not have had the full range of comorbidities present in unselected patient populations. While the majority of studies did not demonstrate an effect of the comorbid condition on outcomes, there is some evidence to support the notion that “less is more” [65] when treating patients with multiple chronic conditions. However, more evidence is needed to inform the care of these patients.

## Supporting Information

Appendix S1 (DOCX)

Checklist S1 PRISMA checklist.
(DOC)
