# Supplementary material for: The Effects of Comorbidity on the Benefits and Harms of Treatment for Chronic Disease: A Systematic Review
Source: PLoS One. 2014 Nov 17;9(11):e112593. doi: 10.1371/journal.pone.0112593 (PMC4234418; doi:10.1371/journal.pone.0112593)
Supplement: Appendix S1 — (DOCX) [file pone.0112593.s001.docx]

APPENDIX: Search strategy

Filter for index conditions:

1 exp heart failure/dt, th or coronary artery disease/dt, th or angina pectoris/dt, th or angina,stable/dt, th

2 exp hypertension/dt, th

3 exp chronic obstructive pulmonary disease/dt, th

4 exp osteoarthritis/dt, th

5 exp hyperlipidemias/dt, th

6 kidney failure, chronic/dt, th

7 exp diabetes mellitus/dt, th

Filter for comorbid conditions:

8 exp heart failure/ or coronary artery disease/ or angina pectoris/ or angina, stable

9 exp hypertension/

10 exp chronic obstructive pulmonary disease/

11 exp osteoarthritis/

12 exp hyperlipidemias

13 kidney failure, chronic

14 exp diabetes mellitus

15 (comorbid* or multimorbid* or multi condition*).mp. or comorbidity/

Filter for study design:

16 exp cohort studies/

17 randomized controlled trial.pt.

18 decision support techniques/

19 16 or 17 or 18

20 su.fs.

21 Guideline Adherence/

22 Program Evaluation/

23 “Drug Utilization Review”/

24 20 or 21 or 22 or 22 or 23

Logic

25 1 and (9 or 10 or 11 or 12 or 13 or 14 or 15)

26 2 and (8 or 10 or 11 or 12 or 13 or 14 or 15)

27 3 and (8 or 9 or 11 or 12 or 13 or 14 or 15)

28 4 and (8 or 9 or 10 or 12 or 13 or 14 or 15)

29 5 and (8 or 9 or 10 or 11 or 13 or 14 or 15)

30 6 and (8 or 9 or 10 or 11 or 12 or 14 or 15)

31 7 and (8 or 9 or 10 or 11 or 12 or 13 or 15)

32 25 or 26 or 27 or 28 or 29 or 30 or 31

33 19 and 32

34 33 not 24

35 limit 34 to “all aged (65 and over)”
